# Short-term transitions in health-promoting lifestyle profiles following first-time percutaneous coronary intervention: a latent profile transition analysis

**DOI:** 10.3389/fpubh.2026.1719320

**Published:** 2026-01-30

**Authors:** Pengli Jiang, Juan Zhang, Wenzhuo Sun, Mengbing Hu, Chan Li

**Affiliations:** Emergency Department, Henan Provincial People's Hospital; Zhengzhou University People's Hospital, Zhengzhou, Henan, China

**Keywords:** behavior change, coronary heart disease, health-promoting lifestyle, latent profile transition analysis, percutaneous coronary intervention

## Abstract

**Background:**

Maintaining a health-promoting lifestyle is essential for long-term cardiovascular risk management in patients undergoing percutaneous coronary intervention (PCI). However, how lifestyle behaviors change before and after first-time PCI, and which factors are associated with these changes, remain insufficiently understood.

**Aims:**

This study aimed to identify patterns and short-term transitions in health-promoting lifestyle behaviors before and after first-time PCI in patients with coronary heart disease (CHD).

**Methods:**

This was a prospective longitudinal study with a 6-month follow-up. A total of 603 patients with CHD from three tertiary hospitals in Zhengzhou were enrolled. Lifestyle profiles were identified at baseline (T1) and at 6 months after PCI (T2) using latent profile transition analysis (LPTA), and multinomial logistic regression was used to examine factors associated with profile transitions.

**Results:**

Three different lifestyles were consistently identified at both time points: Unhealthy, Moderately Healthy, and Healthy. Although some transitions toward healthier profiles were observed between T1 and T2, most participants remained in their initial profile, indicating relative stability over the 6-month period. Higher levels of general self-efficacy and perceived social support significantly increased the likelihood of positive lifestyle changes (*p* < 0.001). In contrast, higher BMI, lower education, and lower income were associated with reduced odds of behavioral improvement (*p* < 0.05).

**Conclusion:**

Lifestyle behaviors among patients undergoing first-time PCI are heterogeneous and show limited change over the first 6 months following the procedure. Psychological and social resources, particularly self-efficacy and social support, are important correlates of favorable lifestyle transitions and may represent useful targets for post-PCI care and rehabilitation.

## 1 Background

Coronary heart disease (CHD) is an acknowledged primary global public health concern. According to the Global Burden of Disease (GBD) study in 2019, the number of prevalent and incident cases reached 197.21 million and 21.20 million, respectively (1). In recent decades, percutaneous coronary intervention (PCI) has become one of the most commonly performed procedures for revascularization in patients with CHD, especially in those with acute coronary syndromes (2). While PCI effectively restores myocardial perfusion and relieves symptoms such as chest pain and breathlessness, it cannot fundamentally address the lifestyle-related risk factors—such as tobacco use, lack of exercise, unhealthy diet, and poor stress management, all of which contribute to the occurrence and development of coronary heart disease (3).

Given this situation, international clinical guidelines always emphasize the necessity and importance of secondary prevention by comprehensive lifestyle changes following PCI (4). Keeping a health-promoting lifestyle has been proven to lower the risk of recurrent cardiovascular events, improve quality of life, and extend the survival time (5–7). Health-promoting behaviors include participating in regular physical activities, maintaining a heart-healthy diet, adhering to medication, managing stress, and avoiding the use of addictive substances (8). However, although the importance of these behaviors has been proven and recognized, for many patients, maintaining them remains a significant challenge, due to a combination of physical limitations after PCI, psychological barriers such as low motivation or fear of overexertion, insufficient knowledge or skills, limited access to resources, and lack of social or family support (9). Studies show that the proportion of patients with heart diseases who insist on a healthy lifestyle is very low, and after the initial intervention, these proportions will further decline over time (10).

According to Bandura’s Social Cognitive Theory (11–13), changing of health behavior is the result of dynamic interactions between personal factors, environmental influences, and behavioral processes. For the personal factors, previous studies have found that ethnicity (14), income (14), comorbidities (14), and education level (15); for environmental influences, Cheng’s study has revealed that the cultural background of patients and the family support could affect the changing of lifestyle (16). In addition to these factors. Individuals with higher levels of self-efficacy are more likely to engage in health-promoting activities, persist when encountering barriers, and recover from setbacks, whereas those with lower self-efficacy tend to disengage more easily from behavior change efforts (17).

Moreover, most existing studies utilized various cross-sectional approaches or focused on single behaviors rather than investigating the dynamic, multidimensional nature of lifestyle changes over time (10, 18). The longitudinal changing in health-promoting lifestyles before and after first-time PCI is not well understood. In particular, there is a lack of hard evidence capturing the transitional patterns and latent subgroups of lifestyle behaviors within this specific population. Understanding these behavioral longitudinal changes is crucial for the development of precise, stage-specific interventions that align with patients’ readiness to change and personalized needs (19).

Latent profile transition analysis (LPTA) is an extension of latent profile analysis (LPA), providing a new perspective on examining changes in unobserved subgroups across multiple time points. LPTA had been used some studies related to lifestyle among different populations, for example, Savage et al. (20) used LPTA identified that four distinct healthy lifestyle profiles were existed in UK university students, including active and highly stressed, less active and well-adjusted, inactive and stressed, and highly active and well-adjusted groups; Janda et al. (21) discovered four activity patterns among the adolescent population in the Czech Republic: including the active sports, active screeners, poor sleeper, and average groups. By identifying different lifestyle profiles and tracking transitions between them, LPTA allows researchers to figure out underlying mechanisms that influence behavioral transformation and maintenance. This method has significant potential for identifying the changing patterns of health promotion strategies among CHD patients undergoing PCI.

Therefore, the present study aims to investigate the longitudinal changes in health-promoting lifestyles among patients undergoing first-time PCI using latent transition analysis. By identifying distinct patterns and their influence factors, this study seeks to provide persuasive evidence to guide future targeted interventions and optimize the long-term management of CHD through behavior-focused secondary prevention.

## 2 Methods

We used the STROBE Checklist for a more rigorous study design and improved article quality.

### 2.1 Study design and population

This was a prospective, longitudinal, descriptive study, approved by the Ethical Committee of Zhengzhou University (ZZUIRB2024-23). All methods were carried out in accordance with relevant guidelines and regulations (Declaration of Helsinki). A random cluster sampling approach was employed during data collection: three tertiary (Level A) general hospitals were randomly selected from the list of eligible tertiary hospitals in Zhengzhou using a computer-generated random sequence.

Participants were followed up at 6 months post-discharge. This follow-up interval was pre-specified at the study design stage to capture early post-PCI behavioral adjustments while minimizing loss to follow-up and recall bias. A 6-month interval is commonly used in cardiac rehabilitation and secondary prevention research as a balance between allowing sufficient time for behavior change to occur and maintaining participant retention (22, 23).

A total of 603 eligible patients were consecutively recruited between September 1 and December 31, 2024, and each participant was followed for 6 months from baseline. The inclusion criteria were as follows: (a) clearly diagnosed coronary heart disease (CHD); (b) aged ≥ 18 years old; (c) able to read and write independently; (d) first accepted percutaneous coronary intervention; (e) willingness to participate in this study. The exclusion criteria included: (a) affliction with severe pulmonary, neurological, musculoskeletal, or psychiatric disorders and tumors; (b) bedridden patients; (c) participation in other studies. Participants who inpatients in these selected hospitals were recruited if they met all inclusion criteria and had none of the exclusion criteria.

Three registered nurses with prior research experience were responsible for data collection. They received standardized training provided by the research team prior to study initiation, which included study objectives, eligibility criteria, informed consent procedures, questionnaire administration, and data quality checks. The in person training lasting approximately 2 h. During the data collection period, the principal investigator conducted regular checks of completed questionnaires to ensure completeness and consistency. The procedures could help to reduce the bias during data collection.

A pilot study was conducted 2 weeks prior to formal data collection to assess the feasibility and clarity of the study procedures and questionnaires. Ten patients who met the inclusion criteria were recruited from one participating hospital. These participants completed the questionnaires and provided feedback on item clarity and survey burden. No major difficulties were identified, and only minor wording adjustments were made before the main study commenced. Data from the pilot study were not included in the final analysis.

The baseline data collection was on the first day after patients’ first PCI (T1), requiring them to recall their last month lifestyle and complete the survey. The follow-up data collection was 6 months after discharge (T2), which was selected based on evidence from previous longitudinal studies indicating that patients’ lifestyle behaviors and functional recovery tend to stabilize after approximately 6 months following PCI, whereas the initial post-discharge period is characterized by more pronounced and unstable changes (22, 23). Positioning the follow-up at 6 months therefore allows for the assessment of early post-PCI lifestyle transitions after the acute adjustment phase, while avoiding measurement during a period of rapid fluctuation. On the baseline data collection day, informed consent was obtained from eligible participants. The investigators used a face-to-face, one-on-one data collection approach, utilizing a translated Chinese version of the instruments. Survey sites were located in each patients’ ward rooms to minimize interference. And the follow-up data collection was carried out by phone. Also, interviewers provided standard explanations for some questionnaire items participants found challenging and ensured their completeness.

### 2.2 Measures

#### 2.2.1 Assessment of covariates

Demographic data included age, gender, income, body mass index, and educational level. The income was obtained from the face to face survey, while the remainders were extracted from the patient’s medical records. Age and body mass index (BMI) were treated as continuous variables, while educational level, gender, and income were categorical variables. Educational attainment was categorized as junior high school or below versus senior high school or above, reflecting a meaningful distinction in the Chinese education system between compulsory education and higher levels of schooling. Monthly per capita income was dichotomized as <3,000 RMB versus ≥3,000 RMB based on local socioeconomic benchmarks and common practice in Chinese public health research to differentiate lower- and higher-income groups.

Comorbidity burden was assessed using the Charlson Comorbidity Index (CCI), which is a validated weighted index that predicts mortality and health outcomes based on the presence and severity of predefined comorbid conditions (24). CCI ranges from 0 upward, with higher scores indicating greater comorbidity burden. Each participant’s CCI score was calculated from medical records at baseline, with higher scores indicating a greater burden of comorbidity.

Participants’ self-efficacy was assessed by the General Self-efficacy Scale (GSES), developed by Schwarzer and Jerusalem (25). It was later translated and revised by Caikang Wang et al. (26) and the results in Chinese populations showed good validation. It is a 10-item psychometric scale that rates optimistic self-beliefs about the ability to cope with difficult situations in life. Each item is awarded a four-point (1–4), represented “Not at all true,” “Hardly true,” “Moderately true,” and “Definitely true,” respectively. Total score ranges from 10 to 40. Higher total scores mean a stronger ability to cope with difficult situations in life. Cronbach’s *α* of the scale in the present sample was 0.893 for T1 and 0.877 for T2.

Perceived social support was assessed by the Perceived Social Support Scale (PSSS), introduced by Blumenthal et al. (27) and compiled by Zimet et al. (28). This measure has been widely used to measure the perceived social support from three sources: family, friends, and significant others. Huang et al. (29) translated it into Chinese and proved it to have good validity and reliability. This scale included 12 items that asked participants to rate on a 7-point Likert scale ranging from 1 (very strongly disagree) to 7 (very strongly agree). Total score ranges from 12 to 84. Higher scores represent higher levels of perceived social support. Cronbach’s *α* of the scale in the present sample was 0.901 and 0.865 for T1 and T2, respectively.

#### 2.2.2 Outcome ascertainment

The health-promoting lifestyle profile questionnaire was developed by Walker et al. (8) and was translated into Chinese by Teng et al. (30), comprised 52 items assessing six dimensions of behavior: health responsibility (nine items), physical activity (eight items), nutrition (nine items), spiritual growth (nine items), interpersonal relations (nine items), and stress management (eight items). The questionnaire was widely used in patients with various chronic diseases and showed good validity and reliability (31, 32). The total scale reliability of Cronbach’s *α* = 0.852, and the 6-dimensional range had Cronbach’s α from 0.729 to 0.894. Each question has a score of 1 to 4, and the total score ranges from 52 to 208, with the higher the score, the better the health promotion lifestyle.

### 2.3 Statistical analysis

The statistical analysis was carried out in three sequential steps to ensure methodological rigor.

Step 1: Data management and preliminary analyses were conducted using SPSS version 17.0 (IBM Corp., Armonk, NY, United States). Cases with missing data on variables of interest were excluded in this study. To assess potential attrition bias, baseline comparisons were conducted between participants who completed follow-up and those lost to follow-up. Analysis of variance (ANOVA) was applied to examine group differences in continuous variables, while Chi-square tests were employed for categorical variables.

Step 2: Mplus version 8.3 was used to perform both Latent Profile Analysis (LPA) and Latent Profile Transition Analysis (LPTA). LPA was conducted to identify latent subgroups based on the mean scores across six dimensions of health-promoting behaviors at Time 1 (T1) and Time 2 (T2), testing solutions ranging from two to five classes. Model fit was evaluated using multiple fit indices, including the Akaike Information Criterion (AIC), Bayesian Information Criterion (BIC), and Sample-Adjusted BIC (aBIC), with lower values indicating better model fit. Subsequently, LPTA was employed to assess transition probabilities between latent classes from T1 to T2, without adjusting for covariates. Model-estimated profile means were exported from Mplus and visualized for presentation.

Step 3: SPSS 17.0 was further used to assess the effects of covariates on transitions between latent health-promoting lifestyle profiles. In the multinomial logistic regression analyses, the dependent variable was transition status between latent lifestyle profiles from T1 to T2, defined as movement from one latent profile to another or remaining in the same profile. The reference category is stability within the same profile from T1 to T2. The independent variables included psychosocial factors (general self-efficacy and perceived social support), health status (Charlson Comorbidity Index), and sociodemographic characteristics (age, sex, body mass index, educational attainment, and monthly income). Continuous predictors were entered into the regression models in their original units and were not standardized prior to analysis. Specifically, age was modeled per 1-year increase, BMI per 1 kg/m^2^ increase, general self-efficacy per 1-point increase on the 10–40 scale, and perceived social support per 1-point increase on the 12–84 scale. In the multinomial logistic regression analyses, the dependent variable was transition status between latent lifestyle profiles from T1 to T2. Multinomial logistic regression models were fitted to estimate odds ratios (*ORs*) for transitioning from one latent profile to another, relative to remaining in the same profile, in the context of individual-level predictors. An OR less than 1 indicated a lower likelihood of transition to other profiles, while an OR greater than 1 suggested a higher likelihood of transitioning, under the influence of a specific covariate.

## 3 Results

### 3.1 Descriptive statistics

603 participants took part in data collection at Time 1 (T1), but 47 were lost to follow-up. 22 of them withdrawal from the study due to lack of interest; 13 were inability to be contacted after repeated attempts; 12 were diagnosed with other diseases, such as cancer and stroke during the 6 months. There were no statistically significant differences in the covariates between the patients who were lost to follow-up and those who completed the follow-up (see Table 1). More details about the 556 participants can be seen in Table 1.

**Table 1:** Comparison of clinical and sociodemographic characteristics between participants who completed the study and those lost to follow-up.

Variables	participants who completed the study N/% or Mean ± SD	participants who lost to follow-up N/% or Mean ± SD	*t/χ^2^*	*p-*value
Sex			0.723	0.392
Male	406 (73.00)	37 (78.70)		
Female	150 (27.00)	10 (21.30)		
Age	43.18 ± 8.18	42.30 ± 9.15	0.432	0.511
BMI	25.15 ± 3.04	24.42 ± 2.71	2.482	0.118
CCI	0.89 ± 0.95	0.81 ± 0.83	0.680	0.410
Social Support	42.43 ± 16.02	35.77 ± 15.12	0.167	0.683
Self-efficacy	29.25 ± 5.06	30.00 ± 4.06	1.637	0.201
Level of education			1.651	0.199
Junior school and below	243 (43.70)	16 (34.00)		
Junior school and above	313 (56.30)	31 (66.00)		
Average monthly income per capita			0.292	0.589
< 3000CNY	470 (76.70)	73 (76.00)		
≥ 3000CNY	143 (23.30)	23 (24.00)		

Values are presented as mean ± SD or n (%). Independent-sample t tests were used for continuous variables and χ^2^ tests were used for categorical variables. CNY, Chinese Yuan; CCI, Charlson Comorbidity Index; BMI, Body Mass Index.

### 3.2 LPA model selection

Table 2 presents the fit indices for all latent profile models of health-promoting lifestyle behaviors at both T1 and T2, revealing a consistent trend across the two time points. As the number of profiles increased, the AIC, BIC, and aBIC values consistently declined, suggesting an improvement in model fit. All models with 2 and 3 profiles demonstrated entropy values above 0.8, reflecting strong classification accuracy (exceeding 90%). Also, the LMR and BLRT tests for the 4-profile solution yielded *p*-values above 0.05 at both time points, indicating no significant improvement over the 3-profile model. Considering these statistical indicators collectively, the 3-profile model was deemed the most appropriate solution for both T1 and T2.

**Table 2:** Fit indices for latent profile models at baseline (T1) and 6-month follow-up (T2).

Time	Profile	AIC	BIC	aBIC	Entropy	LMR (*p*-value)	BLRT (*p*-value)	Percentage (%)
T1	2	2885.670	2867.35	2869.43	0.878	<0.001	<0.001	29.5/70.5
	**3**	**2145.249**	**2136.742**	**2135.49**	**0.866**	**<0.001**	**<0.001**	**11.6/22.2/66.2**
	4	2143.280	2122.500	2132.04	0.797	0.118	0.122	6.6/15.7/20.825/56.9
T2	2	2847.86	2864.42	2866.52	0.882	<0.001	<0.001	31.2/68.8
	**3**	**2137.45**	**2128.37**	**2127.43**	**0.855**	**<0.001**	**<0.001**	**18.7/24.2/49.3**
	4	2134.48	2122.55	2123.65	0.761	0.078	0.085	5.8/17.3/22.8/54.1

The bold values show the best fit, revealing why we chose the model with three profiles.

### 3.3 Profile characteristics

**Figure 1:**
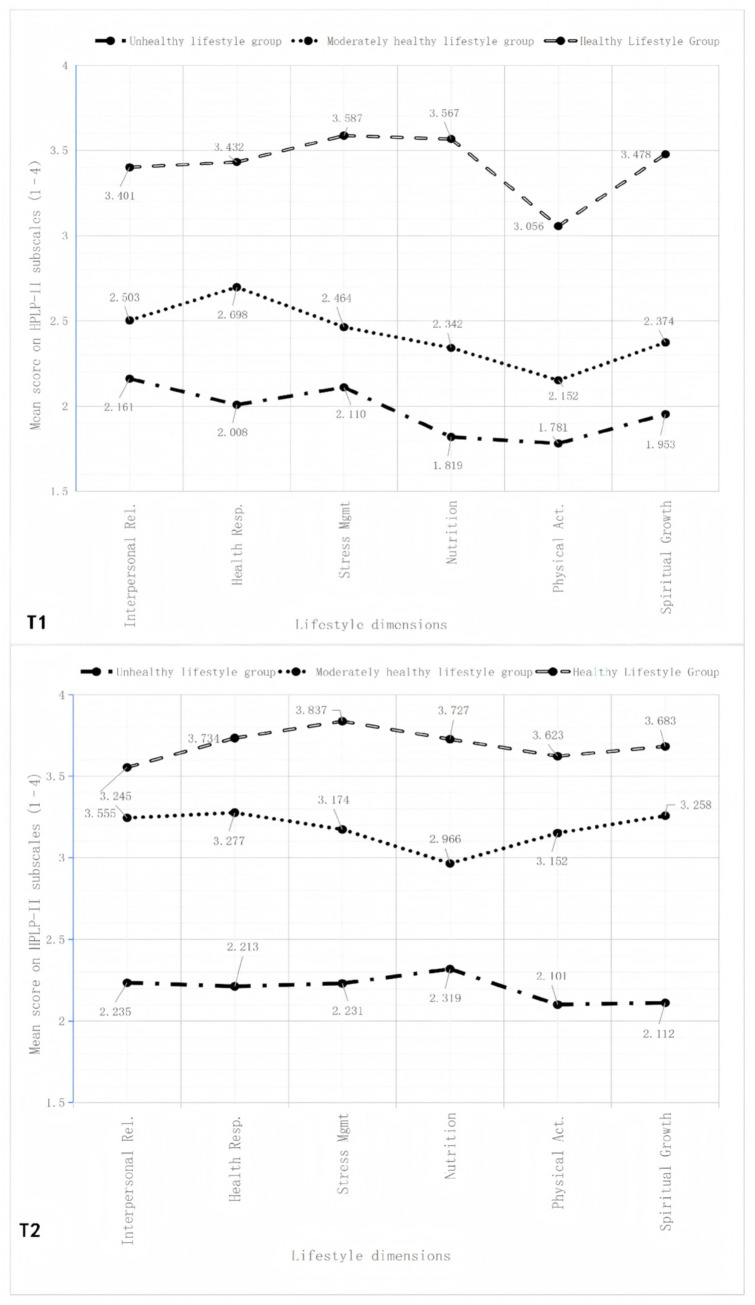
Mean scores of six lifestyle dimensions for each latent profile at baseline (T1) and 6-month follow-up (T2) among 556 patients undergoing first-time PCI.

The estimated trajectories of the three-profile model are illustrated in Figure 1. At Time 1 (T1), the lowest-profile group exhibited mean scores around 2 across the six behavioral dimensions, the intermediate group demonstrated means approximately at 2.5, while the highest-profile group showed mean values exceeding 3. Based on the relative positioning and behavioral patterns, these profiles were labeled as the *Unhealthy Lifestyle Group* (66.2%), *Moderately Healthy Lifestyle Group* (22.2%), and *Healthy Lifestyle Group* (11.6%), respectively.

**Figure 2:**
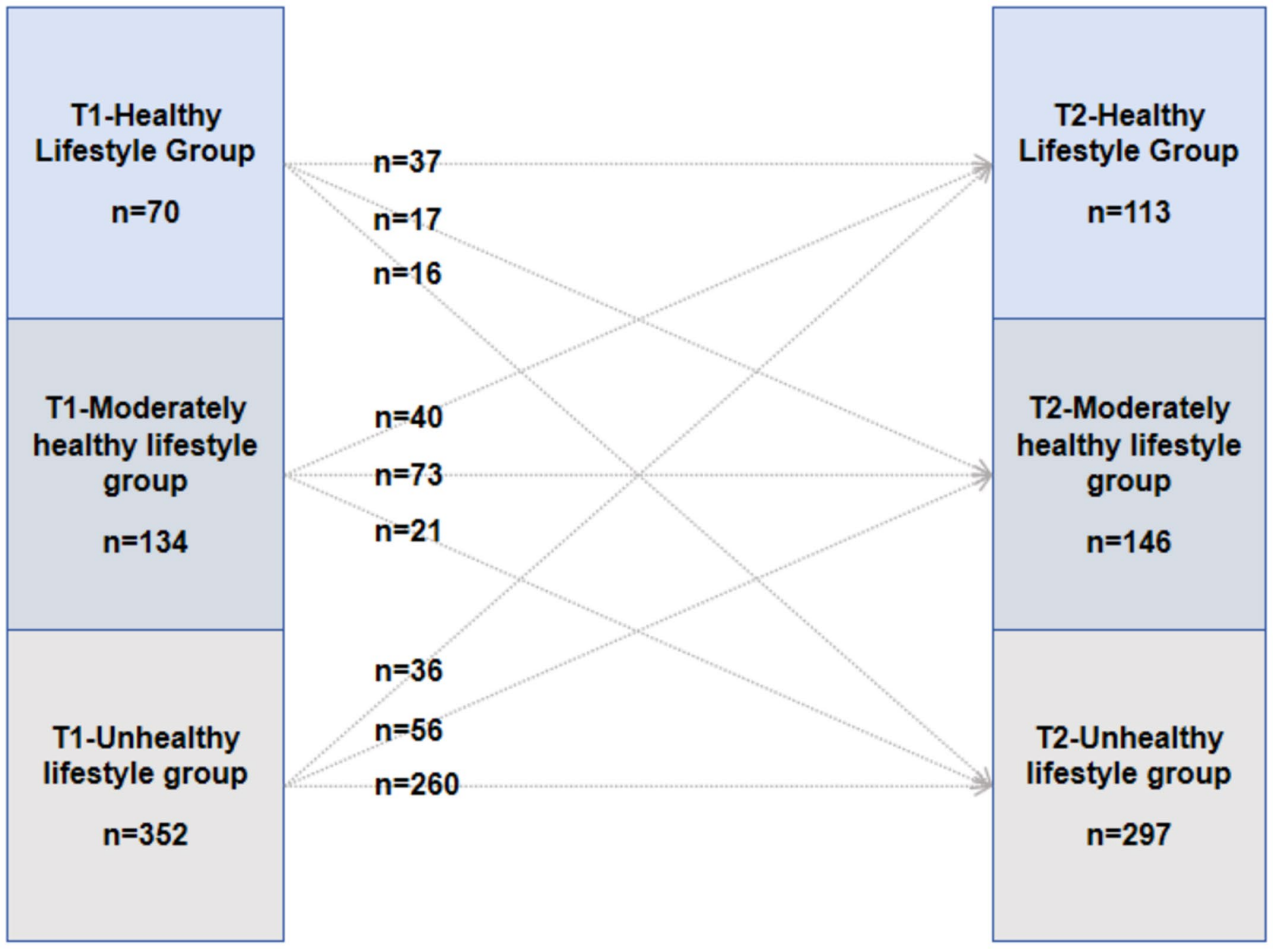
Transitions between latent lifestyle profiles from baseline (T1) to 6-month follow-up (T2).

At Time 2 (T2), a similar three-tiered structure was identified based on the same six dimensions, although the model-estimated mean levels differed from those observed at T1. For descriptive consistency, the same profile labels were retained—namely, Unhealthy Lifestyle Group (49.3%), Moderately Healthy Lifestyle Group (24.3%), and Healthy Lifestyle Group (18.7%). Although the model-estimated mean levels differed between T1 and T2, profiles at each time point were defined based on relative differences rather than a fixed absolute criterion. Therefore, changes in profile proportions should not be interpreted as absolute improvements or declines. The proportions of each profile at both T1 and T2 are shown in Figure 2 and Table 3.

**Table 3:** Transition probabilities between latent lifestyle profiles from baseline (T1) to 6-month follow-up (T2).

Baseline profile (T1)	T2-healthy lifestyle group n (%)	T2-moderately healthy lifestyle group n (%)	T2-unhealthy lifestyle group n (%)	Total
T1-Healthy Lifestyle Group	37 (52.9)	17 (24.3)	16 (22.9)	70
T1-Moderately healthy lifestyle group	40 (29.9)	73 (54.5)	21 (15.7)	134
T1-Unhealthy lifestyle group	36 (10.2)	56 (15.9)	260 (73.9)	352

Values represent the percentage of participants transitioning from each baseline profile (rows) to each follow-up profile (columns).

### 3.4 Covariate effects

To examine the effect of covariates on profile transitions, *ORs* were calculated using stability from T1 to T2 as the reference. As shown in Table 4, self-efficacy and perceived social support were significantly associated with profile transitions. Details are in Table 4.

**Table 4:** Multinomial logistic regression results for predictors of transitions between latent lifestyle profiles from T1 to T2.

Predictors	T1 latent profiles	T2-healthy lifestyle group			T2-moderately healthy lifestyle group			T2-unhealthy lifestyle group		
		*OR*	*p*	*95%CI*	*OR*	*p*	*95%CI*	*OR*	*p*	*95%CI*
Age	T1-Healthy Lifestyle Group	-	-	-	0.958	0.485	0.850–1.081	0.851	0.070	0.715–1.013
	T1-Moderately healthy lifestyle group	1.015	0.597	0.960–1.073	-	-	-	1.087	0.088	0.988–1.197
	T1-Unhealthy lifestyle group	0.958	0.283	0.886–1.036	1.022	0.374	0.947–1.072	-	-	-
Gender (ref = male)	T1-Healthy Lifestyle Group	-	-	-	3.903	0.219	0.444–34.319	9.798	0.101	0.640–150.093
	T1-Moderately healthy lifestyle group	1.446	0.480	0.520–4.016	-	-	-	0.816	0.753	0.229–2.901
	T1-Unhealthy lifestyle group	0.951	0.939	0.265–3.421	0.855	0.708	0.377–1.940	-	-	-
Charlson	T1-Healthy Lifestyle Group	-	-	-	3.569	0.119	0.719–17.707	6.017	0.058	0.941–38.485
	T1-Moderately healthy lifestyle group	0.704	0.338	0.343–1.443	-	-	-	2.182	0.079	0.912–5.217
	T1-Unhealthy lifestyle group	1.363	0.389	0.674–2.760	1.207	0.415	0.768–1.898	-	-	-
BMI	T1-Healthy Lifestyle Group	-	-	-	1.142	0.427	0.823–1.586	1.336	0.204	0.854–2.090
	T1-Moderately healthy lifestyle group	1.294	0.008	1.069–1.566	-	-	-	1.277	0.050	1.000–1.630
	T1-Unhealthy lifestyle group	0.820	0.075	0.660–1.020	0.857	0.035	0.743–0.989	-	-	-
Education Level (ref = Junior school and above)	T1-Healthy Lifestyle Group	-	-	-	3.327	0.348	0.270–40.942	2.569	0.509	0.156–42.235
	T1-Moderately healthy lifestyle group	0.095	<0.001	0.029–0.313	-	-	-	4.357	0.051	0.991–19.162
	T1-Unhealthy lifestyle group	2.621	0.151	0.704–9.761	1.147	0.751	0.492–2.677	-	-	-
Income (ref = ≥ 3000CNY)	T1-Healthy Lifestyle Group	-	-	-	2.225	0.508	0.209–23.713	10.242	0.105	0.616–170.182
	T1-Moderately healthy lifestyle group	2.640	0.100	0.830–8.390	-	-	-	0.297	0.056	0.086–1.033
	T1-Unhealthy lifestyle group	0.172	0.013	0.043–0.687	1.709	0.235	0.705–4.142	-	-	-
Perceived social support	T1-Healthy Lifestyle Group	-	-	-	0.820	0.001	0.727–0.924	0.778	<0.001	0.681–0.889
	T1-Moderately healthy lifestyle group	1.071	0.003	1.023–1.122	-	-	-	0.924	0.001	0.880–0.970
	T1-Unhealthy lifestyle group	1.261	<0.001	1.173–1.355	1.156	<0.001	1.108–1.206	-	-	-
General self-efficacy	T1-Healthy Lifestyle Group	-	-	-	0.873	0.267	0.688–1.109	0.742	0.036	0.561–0.981
	T1-Moderately healthy lifestyle group	1.214	0.032	1.016–1.449	-	-	-	0.909	0.241	0.776–1.066
	T1-Unhealthy lifestyle group	2.656	<0.001	1.828–3.858	1.366	<0.001	1.206–1.546	-	-	-

OR, odds ratio; CI, confidence interval.

From the Unhealthy group, higher self-efficacy increased the odds of shifting to Healthy (*OR* = 2.656, *p* < 0.001) and Moderately Healthy (*OR* = 1.366, *p* < 0.001). From the Moderately Healthy group, higher self-efficacy predicted a shift to Healthy (*OR* = 1.214, *p* = 0.032). Conversely, in the Healthy group, lower self-efficacy increased the risk of decline to Unhealthy (*OR* = 0.742, *p* = 0.036).

Higher perceived social support was associated with lower odds of decline from the Healthier profile (Healthy→Moderately Healthy; *OR* = 0.820, *p* = 0.01; Healthy→Unhealthy; *OR* = 0.778, *p* < 0.001; Moderately Healthy→Unhealthy; *OR* = 0.924, *p* = 0.001;) and higher odds of improvement among participants in lower baseline profiles (e.g., Unhealthy→Moderately Healthy; *OR* = 1.071, *p* = 0.003; Unhealthy→Healthy; *OR* = 1.261, *p* < 0.001; Moderately Healthy→Healthy; *OR* = 1.156, *p* < 0.001).

BMI also mattered: higher BMI increased the odds of moving from Moderately Healthy to Healthy (*OR* = 1.294, *p* = 0.008), but decreased the chance of shifting from Unhealthy to Moderately Healthy (*OR* = 0.857, *p* = 0.035).

Lower education reduced the odds of transitioning from Moderately Healthy to Healthy (*OR* = 0.095, *p* < 0.001), and lower income decreased the likelihood of entering the Healthy group (*OR* = 0.172, *p* = 0.013).

## 4 Discussion

This study applied latent profile transition analysis to describe short-term transitions in health-promoting lifestyle profiles among patients with coronary heart disease undergoing first-time PCI. Three distinct profiles—Unhealthy, Moderately Healthy, and Healthy—were identified at both baseline and 6-month follow-up, and most participants remained in their initial profile over time, indicating relative stability during this period. Modest transitions toward healthier and less healthy profiles were nevertheless observed. In addition, higher self-efficacy and perceived social support were associated with a greater likelihood of transitioning toward healthier profiles, whereas lower educational attainment and lower income were associated with reduced odds of improvement. Associations involving BMI were more variable and appeared to differ by baseline profile.

An important methodological consideration is that the baseline (T1) assessment was conducted after PCI, with participants reporting their lifestyle behaviors retrospectively for the preceding month. This design choice reflects the practical constraints of recruiting patients prior to an acute cardiac event, but it also has implications for interpretation. Immediately after PCI, patients may experience heightened health awareness, emotional impact, and increased motivation to adopt healthier behaviors, a phenomenon often described as a “teachable moment” following acute health events (33). In addition, hospitalization temporarily structures patients’ routines (e.g., regulated diet, smoking restriction, reduced work demands), which may influence both actual behaviors and self-reports (34). As a result, baseline lifestyle reports may be subject to recall bias or social desirability bias, and may not fully reflect patients’ habitual pre-PCI behaviors. This context may partly explain the relatively modest transitions observed over the follow-up period and suggests that changes captured in this study should be interpreted as early post-event adjustments rather than long-term behavioral trajectories.

By applying LPTA, this study captured subgroup heterogeneity and within-person transitions that may be overlooked in traditional regression-based approaches (35). The identification of similar profile structures at both time points suggests a stable underlying pattern of lifestyle differentiation in this population. Although transitions between profiles were observed, their magnitude was modest over the 6-month period. A study conducted in Italy reported larger changes in adherence to diet and exercise during the first 6 months after acute coronary syndrome (10). While direct comparison is limited by differences in measurement and context, this contrast highlights the potential influence of sociocultural environments on lifestyle change. For example, adherence to the Mediterranean diet and population-level physical activity levels are generally higher in Italy than in China (36), which may facilitate behavioral change following acute cardiac events. Nevertheless, even with modest overall transition rates, the present findings provide important insights into heterogeneity in lifestyle adaptation after PCI. In particular, individuals classified in the lowest-profile group at baseline exhibited the highest likelihood of transitioning to higher profiles, indicating a subgroup with substantial potential for change.

In this study, we found that general self-efficacy and perceived social support emerged as significant predictors of health-promoting lifestyle transitions. Higher self-efficacy was associated with a greater likelihood of improvement from Unhealthy or Moderately Healthy profiles toward the Healthy profile, while participants in Healthy lifestyle group with lower self-efficacy was likely to decline toward Unhealthy group. Similar results could be seen in other studies, for example, Simone et al. (37) found that higher self-efficacy could be a relevant motivator for middle-aged people to take part in healthy lifestyle behaviors to reduce the risk of dementia. These findings support Bandura’s Social Cognitive Theory, which emphasizes self-efficacy as a key element of continuous health behavior (11). Changing to a healthy lifestyle, encompassing regular physical activity, a healthy diet, weight management, and smoking cessation, could be seen as challenging for people (16). Individuals with stronger self-efficacy are more confident in tackling challenging tasks during the transition to a healthier lifestyle, and they are more likely to set goals, take action, persevere, and recover from a heart attack and operation (38). Patients with low efficacy feel a lack of motivation and patience for behavioral change, and they tend to give up easily once they encounter obstacles during the switch from an unhealthy lifestyle to a healthy one (39). This finding reminded us that assessing patients’ general self-efficacy is crucial to tailor health promotion interventions effectively. Professionals can provide targeted education, set achievable goals with patients, and use motivational interviewing to build confidence in managing difficult behavioral changes. Encouraging self-monitoring and celebrating small successes may reinforce a sense of control. For patients with low self-efficacy, more specific structured health education and emotional support are need to be provided to reduce discouragement and promote persistence.

Similarly, patients with better perceived social support have increased odds of transitioning to healthier profiles, suggesting that interpersonal and emotional support might play an essential role in promoting and sustaining healthier behavior changing. The result is aligned with the result of Breeman’s study (40), even though the participants in their study were patients with various cardiovascular diseases. According to Social Cognitive Theory (11), interpersonal influences—such as encouragement, role modeling, and emotional reassurance—can enhance patients’ outcome expectations and confidence in their ability to adopt and maintain new healthy behaviors. According to previous study, having support from family members, friends, or significant others may help CHD patients overcome practical and psychological barriers to lifestyle modification (41). Our findings extend this evidence to the post-PCI population, reinforcing the need for nursing interventions that actively engage family members, peers, or community support systems. Former studies (42, 43) have proven that family participation is at the core of health-related decision-making. Customizing support strategies based on the social and cultural background of patients may enhance their effectiveness. Integrating structured support programs into cardiac rehabilitation and post-discharge education can significantly improve behavioral outcomes.

Our findings suggest that BMI may play a complex and context-dependent role in lifestyle transitions. Among participants in the Moderately Healthy profile at baseline, a higher BMI was associated with greater odds of transitioning to the Healthy profile. In contrast, among participants in the Unhealthy profile at baseline, a higher BMI was associated with lower odds of transitioning to a Moderately Healthy profile. This may indicate that individuals with both higher BMI and unhealthy lifestyle patterns face greater physical, psychological, or structural barriers when initiating behavior change. Importantly, these divergent associations should be interpreted cautiously. Prior qualitative research suggests that lifestyle change among individuals with higher BMI is strongly influenced by social and interpersonal factors, such as support from family and friends, indicating that BMI itself is unlikely to be a sole or direct determinant of behavior change (44). Additionally, a systematic review on the factors influencing lifestyle change indicated that the impact of higher BMI on lifestyle change remains unclear (45). These findings raise the possibility of reverse causality (i.e., early behavior change influencing weight rather than weight influencing behavior), as well as the limitation of BMI as a crude indicator that does not capture body composition, fitness, or metabolic health (44, 45). Taken together, BMI in this study may serve as a marker of different motivational or health contexts across baseline profiles, rather than indicating a simple directional effect. This highlights the importance of considering baseline lifestyle patterns when interpreting associations between BMI and behavioral change, and suggests that individuals with higher BMI and unhealthy lifestyle profiles may benefit from more tailored and supportive interventions.

In this study, sociodemographic characteristics such as lower educational level and low income were also proven to be associated with a lower likelihood of transitioning to healthier lifestyle profiles. These findings are similar to previous studies (46, 47) carried out in other developing countries, indicating that individuals with lower socioeconomic status face greater medical information barriers to health behavior change, including limited access to health education, community-based healthy programs, and affordable healthy food or exercise opportunities. Moreover, although financial burden and health insurance coverage were not directly assessed in this study, financial stress and treatment-related costs may further constrain patients’ ability to engage in lifestyle change (48), particularly in healthcare systems without comprehensive insurance coverage, like China, where this study was conducted.

In contrast to some previous studies (49, 50), neither age, nor CCI, nor sex was significantly associated with lifestyle transitions in the present study. That might due to the age range in this cohort was relatively narrow, with most participants being middle-aged, which may have limited the ability to detect age-related differences. Additionally, patients with severe comorbidities were excluded, resulting in restricted variability in CCI and a generally low comorbidity burden. The absence of sex differences may also be related to limited statistical power to detect such effects within the transition model. Although the overall sample size was adequate for identifying latent profiles, the number of participants within specific transition pathways was relatively small, which reduces sensitivity for detecting subgroup differences. In addition, sex effects on lifestyle behaviors are often modest in magnitude, and such effects may be overshadowed by stronger psychosocial predictors, such as self-efficacy and perceived social support, in multivariable models.

Although previous studies have reported higher participation in cardiac rehabilitation and health behaviors among men than women, no sex-related differences were observed in lifestyle transitions in this study. This discrepancy may reflect contextual differences in healthcare delivery and social roles. In the present setting, structured cardiac rehabilitation programs were not routinely available, and all patients received similar standardized discharge education, potentially reducing sex-based differences in access to resources. Moreover, cultural norms regarding family involvement and caregiving may provide women with compensatory social support that offsets lower formal participation in rehabilitation programs. It is also possible that sex differences emerge over longer follow-up periods, whereas the present study focused on early post-PCI transitions.

From a clinical perspective, these findings suggest that psychosocial factors could be more systematically incorporated into post-PCI care and cardiac rehabilitation. General self-efficacy and perceived social support can be used using during the hospitalization. Routine screening could help identify patients who lack social support or have lower self-efficacy and who may be at higher risk of experiencing difficulty initiating or sustaining lifestyle changes. Such assessments could be integrated into existing cardiac rehabilitation programs to support risk stratification and individualized care planning. For example, patients with lower self-efficacy could be offered additional motivational interviewing, goal-setting interventions, or behavioral coaching (51), while those with limited social support could be encouraged to involve family members or peers in rehabilitation sessions or be referred to community-based support resources (52). Evidence from cardiac rehabilitation and secondary prevention studies suggests that tailored, psychosocially informed interventions are more effective than uniform advice alone in promoting sustained lifestyle change and adherence to secondary prevention behaviors (53, 54). In a word, these findings highlight the potential value of moving beyond a one-size-fits-all approach to lifestyle counseling after PCI and adopting a more personalized, psychosocially informed model of secondary prevention.

This study has several limitations that should be acknowledged. First, the follow-up period was limited to 6 months after PCI, which may be insufficient to capture longer-term lifestyle maintenance or delayed behavioral changes. The findings therefore reflect short-term transitions rather than sustained behavioral trajectories. Second, this was an observational study, and causal inferences cannot be drawn regarding the relationships between psychosocial, clinical, and sociodemographic factors and lifestyle transitions. In addition, lifestyle behaviors, self-efficacy, and perceived social support were assessed using self-reported instruments, which may be subject to recall bias and social desirability bias. Third, latent profiles were derived separately at each time point and therefore represent relative classifications within each wave rather than absolute lifestyle standards. As a result, changes in the relative size of profiles across time should not be interpreted as absolute prevalence changes in healthy or unhealthy lifestyles. Fourth, the study was conducted in three tertiary hospitals in a single urban setting in China, which may limit the generalizability of the findings to rural populations, lower-level hospitals, or healthcare systems with different organizational and financial structures. Fifth, although perceived social support was measured, the specific role of family caregivers, caregiving intensity, and household-level dynamics was not directly assessed. Similarly, financial burden, health insurance coverage, and financial toxicity were not measured, and these factors may influence patients’ ability to adopt and sustain lifestyle changes.

## 5 Conclusion

This study provides a nuanced view of how health-promoting lifestyle behaviors evolve in the early months following PCI and highlights that meaningful change is neither uniform nor automatic. While some patients demonstrate improvements, many remain stable, and others experience difficulty adopting healthier patterns, underscoring the heterogeneity of behavioral adjustment after PCI. These findings emphasize that effective secondary prevention requires more than standardized advice and should instead adopt a person-centered approach that considers patients’ psychological resources, social contexts, and structural constraints. Supporting patients’ confidence to change, strengthening their social support systems, and addressing socioeconomic barriers may be particularly important for facilitating sustainable lifestyle improvement after PCI. Together, this work underscores the need for integrated, tailored, and context-sensitive strategies to promote long-term cardiovascular health.

## Data Availability

The raw data supporting the conclusions of this article will be made available by the authors, without undue reservation.
